# Safeguarding the Caribbean's future: making the case to research the direct and indirect impacts of climate change on youth mental health and wellbeing

**DOI:** 10.3389/fpubh.2023.1322831

**Published:** 2023-12-13

**Authors:** Jaclyn Holdsworth, Shelly-Ann Hunte, Kershelle Barker, Jonathan Sherin, Mala Rao, Sandeep B. Maharaj

**Affiliations:** ^1^Queen Elizabeth Scholarship Program, McMaster Health Forum, Hamilton, ON, Canada; ^2^Caribbean Centre for Health Systems Research and Development, The University of the West Indies, St. Augustine Campus, St. Augustine, Trinidad and Tobago; ^3^Evidence Synthesis, Caribbean Centre for Health Systems Research and Development, The University of the West Indies, St. Augustine Campus, St. Augustine, Trinidad and Tobago; ^4^Healthy Brains Global Initiative, Los Angeles, CA, United States; ^5^Ethnicity and Health Unit, Department of Primary Care and Public Health, Imperial College London, London, United Kingdom; ^6^School of Pharmacy, The University of the West Indies, St. Augustine Campus, St. Augustine, Trinidad and Tobago

**Keywords:** Small Island Developing States, Caribbean, mental health, climate change, youth

## Abstract

This article makes a call for attention to paid on the development of a research agenda for studying the impact of climatic events on youth mental health in the Caribbean. It details the climate injustices that the region faces and the potential mental health problem which can arise from climatic events. It makes a call for interdisciplinary research and a multi stakeholder approach to dealing with this potential issue.

This paper seeks to outline the case for developing a research agenda for measuring the impact, both direct and indirect, on youth mental health in the Caribbean region. The challenges of climate change within the English Speaking Caribbean are compounded by the Region's experiences with the structural violence of colonialism (1–3). Scholars have argued for decades that climate change is an inherently racist crisis, with the burden being disproportionately felt by tropical and sub-tropical communities that were colonized and racialized (3). Colonialism's consequences live on in many tangible ways, including in many Caribbean states' cash crop practices and poor infrastructure (1). Power imbalances continue in international trade agreements, climate financing, development interventions, and more (3). Many nations have reduced capacities to address climate change explicitly because the Global North extracted resources, successively through colonialism, imperialism, and neocolonialism (3). This left the nations in poverty with little capacity to overcome their socioeconomic deprivation.

The Caribbean is entirely composed of Small Island Developing States (SIDS) and is one of the regions most vulnerable to climate change (floods, storms, and droughts) (1, 4–7). The Atlantic basin, where the Caribbean region is located, exposes most Caribbean islands to hurricanes (5, 7). Between 1980 and 2014, the Caribbean experienced 70 named tropical cyclones. The frequency increased over the past decade, with three category-five hurricanes between 2017 and 2019 (5). Caribbean SIDS are also already experiencing coastal erosion, rising sea levels, stronger extra-tropical and tropical cyclones, rising air and sea surface temperatures, and altered rainfall patterns (1, 5). Approximately 70% of the Caribbean's population resides in coastal communities (5, 7) and ocean acidification, altered weather, freshwater salinification, invasive species etc., are projected to significantly change local ecosystems (1). Attribution science shows that an increasing number of natural disasters are expected to be directly tied to climate change (4).

The effects of climate change are impacting the cultural survival, livelihoods, and wellbeing of island communities (1). There is evidence to indicate that people who are exposed to climate change events may develop a number of mental health issues, including post-traumatic stress disorder, depression, and anxiety (1, 6–16). The pathways for these impact on one's mental health have been found to be both direct and indirect (17). The negative impacts on the health, including the mental health, of SIDS populations are projected to worsen (1, 7). Several characteristics of Caribbean SIDS impede their ability to effectively respond to and prepare for climate change. SIDS are exposed to various stresses, both climatic and non-climatic, due to their specific characteristics, which include their physical size, small populations, susceptibility to natural disasters, geographical isolation, economic vulnerability, and low adaptive capacity (1, 5). The Caribbean region's high level of public debt reduces the region's resilience, even as geographical and demographic features increase populations' vulnerability to changing climate (1, 5). The World Bank estimates that the damage caused by climate-related and earth-related hazards is $12.6 billion per year for the Caribbean (18).

There have been some successes however within the region with regards to climate change, ICT for example has been leveraged for disaster planning and response (19). When it comes to building plans of action there are 89 Caribbean adaptation planning documents (20). However, the challenge remains, utilization, linkages between plans and monitoring and evaluation of strategies implemented.

## Mental health and youth issues faced in Caribbean SIDS

Internationally and within the Caribbean region, many youths lack perceived and/or actual political or economic power over the climate crisis (5, 14). Youth are feeling worry, anger, powerlessness, fear, guilt, anxiety, and helplessness in response to the climate crisis (6, 10, 14). As a result of their lack of coping skills, young people are thought to be more susceptible to the harmful consequences of climate change. Youth's ability to cope with stress and uncertainty is affected by their ongoing neural and cognitive development (6). Emerging evidence suggests that climate anxiety and related phenomena such as ecological grief may have substantial adverse effects on young people's mental health (4, 6). It has been reported that Caribbean children are among the most vulnerable populations to climate change (10). Young activists are witnessing increasing natural disasters and dealing with the wellness implications, even as mental wellbeing appears to be a low healthcare priority in many SIDS (1).

Young people's perspectives have been neglected by policymakers and researchers globally, to the detriment of both the environment and youths' wellbeing (21). According to the *Status of the World's Children* 2021 study (22), almost 16 million teenagers in Latin America and the Caribbean, aged 10–19, are experiencing a mental disorder. The youth mental health crisis has recently been made worse by the coronavirus disease 2019 (COVID-19) pandemic (23). Exposure to climate-related disasters has been found to exasperate existing mental health conditions in youth (10).

Systems for improving mental health, which include organizations and resources, should be adaptable to regional needs. Challenges with the mental health systems include restricted access to specialized mental health practitioners and facilities (24). Small-island settings may encounter difficulties meeting international mental health standards. This would include recovery and care, such as the compulsory treatment and detention and the long-term, intersectoral rehabilitation services that are needed to meet the complex health, social, and economic needs of persons with chronic mental disorders (24). A lack of available and accessible mental health services may increase inequalities in mental health outcomes within SIDS. These and other challenges are encapsulated in Table 1, with potential solutions identified.

**Table 1:** Key and synthesized findings.

**Topic**	**Problem**	**Solution**	**Outcome**	**Challenges**
Research on youth mental health and climate change using a one health approach.	Lack of literature in the field.	Development of interdisciplinary research teams.	Enhance understanding of the problem.	Integrating the teams.
Decolonizing the youth mental health and climate change agenda.	SIDS have historically been on the wrong side of the Global Agenda, due to colonialism, imperialism, and neocolonialism.	During vulnerable groups, lived experience and youth to the table in research and policy.	Bringing voices that were previously unheard but affect by the crisis to have inputs.	Finding the appropriate voices.
Stakeholder engagement.	Currently the stakeholder, civil society, research and government are Siloed.	Engagement of the relevant networks for research, policy, and operationalization of the youth mental health and climate change agenda.	The implementation of the Caribbean Youth Mental Health and Climate Agenda will be rapid due to synergies developed.	Getting persons, who are already pressed for time to the table and changing the culture.

Youth in Caribbean SIDS have been identified as being vulnerable to mental health challenges related to climate change (2, 10). However, there is a dearth of research on the topic. There are fewer published articles about climate and health in the Caribbean when compared to other regions (7). Mental health in the context of climate change has been especially absent within SIDS-related research, policy, and action. The available literature on climate change and Caribbean youth's mental health identifies a need for research investigating the impacts of climate changes on youths' physical, psychological, social, and emotional wellbeing (1, 4, 14, 25). Future work in the field needs to be interdisciplinary (4); decolonizing and informed by lived experiences (3); and collaborative using a One Health approach (7). Engagement of the relevant networks of researchers, civil society, and Ministries is needed to advance the Caribbean's health and climate change agenda (7).

## Data availability statement

The original contributions presented in the study are included in the article/supplementary material, further inquiries can be directed to the corresponding author.

## Author contributions

JH: Conceptualization, Writing—original draft. S-AH: Writing—original draft. KB: Writing—review & editing. JS: Writing—review & editing. MR: Writing—review & editing. SM: Conceptualization, Writing—review & editing.
